# Nitric Oxide and Salicylic Acid Regulate Glutathione and Ethylene Production to Enhance Heat Stress Acclimation in Wheat Involving Sulfur Assimilation

**DOI:** 10.3390/plants11223131

**Published:** 2022-11-16

**Authors:** Faisal Rasheed, Iqbal R. Mir, Zebus Sehar, Mehar Fatma, Harsha Gautam, Sheen Khan, Naser A. Anjum, Asim Masood, Adriano Sofo, Nafees A. Khan

**Affiliations:** 1 Plant Physiology and Biochemistry Laboratory, Department of Botany, Aligarh Muslim University, Aligarh 202002, India; 2 Department of European and Mediterranean Cultures: Architecture, Environment, Cultural Heritage (DiCEM), University of Basilicata, 75100 Matera, Italy

**Keywords:** heat tolerance, nitric oxide, photosynthesis, salicylic acid, wheat

## Abstract

Phytohormones have a role in stress adaptation. The major mechanism underlying the role of exogenously-sourced nitric oxide (NO; as sodium nitroprusside, SNP: 50.0 µM) and salicylic acid (SA; 0.5 mM) in the presence of 2.0 mM SO_4_^−2^ was assessed in heat stress (HS; 40 °C for 6 h daily for 15 days) tolerance in wheat *(Triticum aestivum* L. cv. HD-3226). The cultivar HD-3226 possessed high photosynthetic sulfur use efficiency (p-SUE) among the six cultivars screened. Plants grown under HS exhibited an increased content of reactive oxygen species (ROS; including superoxide radical and hydrogen peroxide) and extent of lipid peroxidation with a consequent reduction in photosynthesis and growth. However, both NO and SA were found to be protective against HS via enhanced S assimilation. Their application reduced oxidative stress and increased the activity of antioxidant enzymes. NO or SA supplementation along with S under HS recovered the losses and improved photosynthesis and growth. The use of SA inhibitor (2-aminoindane-2-phosphonic acid; AIP) and NO scavenger (cPTIO) confirmed that the mitigating effects of SA and NO involved induction in S assimilation.

## 1. Introduction

Achieving global food security has been one of the major aims of the current programme on crop plant research [1]. Notably, crop plant health and productivity are largely decided by several climatic factors, including temperature [2]. Ironically, the global temperature has increased by 0.87 °C during the period from 2006 to 2015 and is likely to increase further by above 1.5 °C by the end of the 21st century [3]. In particular, the elevated temperature is one of the major abiotic stresses that hamper plant growth and productivity worldwide [4,5]. Furthermore, high temperature exposure can cause other abiotic stresses such as drought and salinity and also affect plant growth by shifting the normal morphological, biochemical and physiological changes towards stressed conditions [6]. A significant imbalance between oxidants (reactive oxygen species, ROS; including superoxide radical and hydrogen peroxide; H_2_O_2_) and antioxidants, the damage in the structure of proteins, disturbed translation, inactivated major enzymes, elevated membrane damage, and also retarded cell divisions and DNA damage have been reported in plants under elevated heat stress (HS) [7,8].

Wheat (*Triticum aestivum* L.) is one of the major staple crops consumed to provide carbohydrate and proteins in diets globally [9]. *T. aestivum* has been cultivated widely and provides a significant amount of protein per gram (12–15%) when compared with rice and maize [10]. Additionally, the cultivation of *T. aestivum* may become reduced with the rise in temperature of 2 °C in temperate and tropical areas [11]. Climate change modelling research also discovered a 6% decrease in *T. aestivum* output, which is approximately equivalent to a potential 42 °C decrease [10,12]. Thus, there is an urgent need to obtain more insights into elevated HS impacts, and also obtain a clear understanding of the adoption of sustainable approaches for improving plant health and yield under increasing inevitable changes in climatic factors, including temperature.

Plants are endowed with an antioxidant defense system comprising enzymatic (ascorbate peroxidase, APX; catalase, CAT; glutathione reductase, GR; glutathione peroxidase, GPX; peroxidase, POD; and superoxide dismutase, SOD) and non-enzymatic (ascorbate, AsA; reduced glutathione, GSH; proline, carotenoids, and flavonoids) antioxidants [13]. Notably, concerted efforts have been made to strengthen the antioxidant defense system through several exogenously-applied mineral nutrients (such as sulfur, S; phosphorus, P; potassium, K; calcium, Ca), phytohormones (such as abscisic acid, ABA; and salicylic acid, SA) and signaling molecules (such as nitric oxide, NO) [4,5,14,15,16].

Among the key mineral nutrients, S plays an important role in improving a plant’s antioxidant mechanisms during various kinds of abiotic stresses [17]. It is a major constituent of important proteins involved in the regulation of metabolism from the seedling stage to the maturity of plants [18]. Additionally, a crosstalk exists between the S-containing compounds (such as GSH, -SH) and other biological active compounds such as phytohormones, enzymes, polyamines and nutrients, which helps provide stress tolerance in plants by strengthening the ROS-scavenging system and improving antioxidant defense [5,13,18,19,20]. A plant growth regulator of free radical gaseous nature, NO (sodium nitroprusside; SNP as NO source) is involved in different plant functions at the molecular level [21]. NO has been found to be a key regulator in regulating physiological responses such as seed germination [22], plant growth, gravitropic responses [23], maturation and senescence [24]. Elevation in the accumulation of NO was reported to help plants to acclimatize under high temperature stress [21,25,26]. Furthermore, the probability of NO release as a generalized stress response has been ruled out, and its functional specificity has been confirmed by scavenging endogenous NO levels by 2-4-carboxphenyl-4,4,5,5-tetramethylimidazoline-1-oxyl-3-oxide (cPTIO) that excluded its beneficial effects in HS [26,27]. Having a phenolic nature, SA is widely involved in the array of growth, physiological and developmental processes and also plays a major role in the direct or indirect signaling responses against biotic and abiotic stresses [18]. Moreover, SA improves photosynthetic functions, nutrient uptake and their assimilation, proline content and osmotic concentration, and combats elevated ROS-caused consequences by strengthening antioxidant defense mechanisms [17,18,28].

Recent reports are available on the coordinating role of NO, N and S (and ethylene) [16] and also on the mechanistic elucidation of SA- and S-induced defense systems, and N metabolism in salinity-exposed test crop plants [14]. However, crosstalk between NO and S for the improved tolerance of plants to HS has been little explored [5]. Given the above, this study hypothesized that the outcomes of crosstalk between NO, SA and S might counteract the major impact of HS in *T. aestivum*. The set of parameters aimed to dissect the role of SA, NO and S in protecting *T. aestivum* against HS.

## 2. Results

### 2.1. Screening of T. aestivum Cultivars for HS Tolerance

In the first experiment, six *T. aestivum* cultivars (HD-3059, HD-3090, HD-3226, HD-3237, HD-3271 and HI-1620) were assessed for their HS tolerance (Table 1). Compared to their respective controls, HD-3059, HD-3090, HD-3226, HD-3237, HD-3271 and HI-1620 exhibited decreases in plant dry mass by 31.4, 32.6, 26.0, 27.4, 29.7 and 29.9%, respectively. Decreases of 31.6, 35.4, 22.6, 25.5, 27.2 and 29.8% were exhibited for the net photosynthetic rate and of 47.4, 50.3, 52.3, 52.9, 54.4 and 52.9% in photosynthetic SUE (p-SUE) in these cultivars, respectively, as compared to their respective controls. The screened cultivars demonstrated p-SUE, plant dry mass and net photosynthetic rates in the order: HD-3226 > HD-3237 > HD-3271 > HI-1620 > HD-3059 > HD-3090 (Table 1).

### 2.2. Effect of NO, S and SA on Growth Parameters and Photosynthetic Characteristics under HS

In the highest p-SUE-exhibiting *T. aestivum* cultivar HD-3226, HS decreased leaf area by 32.3% and plant dry mass by 45.9% in comparison to the control plants (Table 2). Individual applications of S, SA and NO significantly (*p* < 0.05) enhanced these growth parameters. However, combined applications of SA + S and NO + S maximally increased plant dry mass by 35.6 and 32.1% and leaf area by 53.8 and 46.5%, respectively, when compared to the control. Under HS, individual applications of S, SA and NO elicited a small but significant increase in the plant dry mass and leaf area compared to control plants. Furthermore, plants receiving NO + S and SA + S under HS showed the maximum alleviation of HS and increased plant dry mass by 28.7% and 26.4%, and leaf area by 44.5% and 38.8%, respectively, compared to the control plants (Table 2).

When compared to plants grown under control conditions, HS lowered net photosynthesis by 46.7%, stomatal conductance by 27.5%, intercellular CO_2_ concentration by 37.9%, SPAD value by 30.8% and F_v_/F_m_ by 22.5% (Table 2). Individual applications of S, SA and NO under non-stress conditions enhanced the examined photosynthetic parameters, while combined applications of NO + S and SA + S boosted them the most. Furthermore, the use of NO with S and SA with S substantially decreased the effects of HS on photosynthetic parameters. The treatment of NO with S under HS maximally enhanced net photosynthesis by 33.6%, stomatal conductance by 19.8%, intercellular CO_2_ concentration by 31.6%, SPAD value by 22.2% and F_v_/F_m_ by 17.5% (Table 2).

### 2.3. Application of NO, S and SA Reduced H_2_O_2_ and Thiobarbituric Acid Reactive Substances (TBARS) Contents under HS

**Figure 1:**
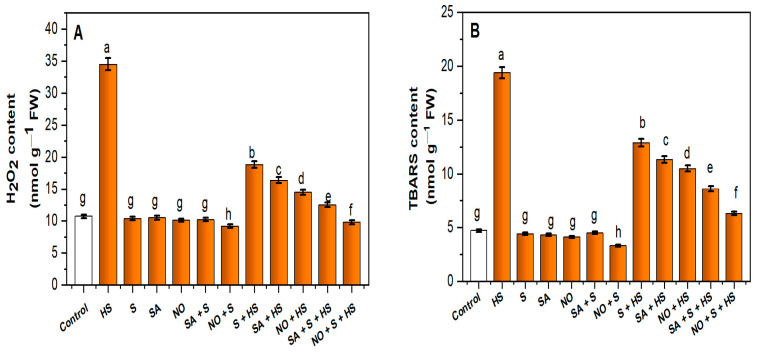
Content of (**A**) hydrogen peroxide (H_2_O_2_) and (**B**) thiobarbituric acid reactive substances (TBARS) in wheat (*Triticum aestivum* L.) cv. HD-3226 treated with 0.5 mM salicylic acid (SA) and/or 50.0 µM nitric oxide (NO)/2.0 mM SO_4_^−2^ (S) individually or in combination in presence or absence of heat stress (HS; 40 °C for 6 h daily for 15 days) at 30 days after germination. Data are presented as treatment mean (*n* = 4). Data followed by the same letter are not significantly different by the LSD test at *p* ≤ 0.05. FW, fresh weight.

Heat exposure considerably increased oxidative stress, which was associated with high H_2_O_2_ (+224.4%) and TBARS (+312.7%) content when compared to the control (Figure 1A,B). The individual treatments of SA, NO and S under HS reduced oxidative stress in terms of H_2_O_2_ by 70.4, 73.3 and 45.5%, and TBARS content by 76.8, 82.9 and 33.5%, respectively, in comparison to only HS-treated plants. Under combined application, SA + S lowered the HS-induced H_2_O_2_ and TBARS content by 63.7 and 55.6%, while reductions recorded under NO + S treatment were 71.5 and 67.5%, respectively, in comparison to HS-treated plants.

### 2.4. Application of NO, S and SA Stimulated Antioxidant Enzyme Activity and S Assimilation under HS

**Figure 2:**
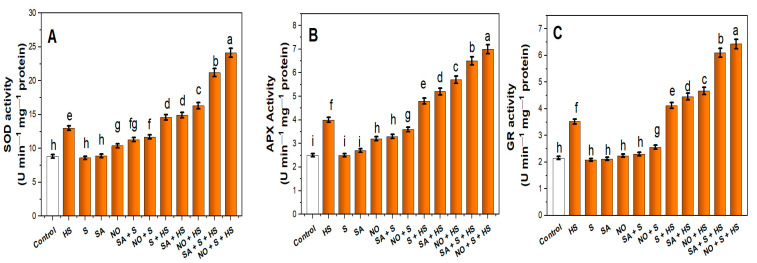
Activity of (**A**) superoxide dismutase (SOD), (**B**) ascorbate peroxidase (APX) and (**C)** glutathione reductase (GR) in wheat (*Triticum aestivum* L.) cv. HD-3226 treated with 0.5 mM salicylic acid (SA) and/or 50.0 µM nitric oxide (NO)/2.0 mM SO_4_^−2^ (S) individually or in combination in the presence or absence of heat stress (HS; 40 °C for 6 h daily for 15 days) at 30 days after germination. Data are presented as treatment mean (*n* = 4). Data followed by the same letter are not significantly different by the LSD test at *p* ≤ 0.05. FW, fresh weight.

HS increased the activity of antioxidant enzymes such as SOD, APX and GR by 47.3%, 60.2% and 63.7%, respectively, compared to the control plants (Figure 2A–C). The application of SA, NO or S to HS-treated plants enhanced SOD, APX and GR activity when compared to only HS plants. Among SA, NO and S, NO supply more prominently increased the activity of SOD by 1.2-times, APX by 1.4-times and GR by 1.3-times compared to HS-treated plants. Moreover, the combined treatment of SA + S and NO + S further stimulated the increase in activity of SOD by 1.4- and 1.8-times, APX by 1.6- and 1.7-times, and GR by 1.6- and 1.9-times, respectively, relative to HS-exposed plants.

**Figure 3:**
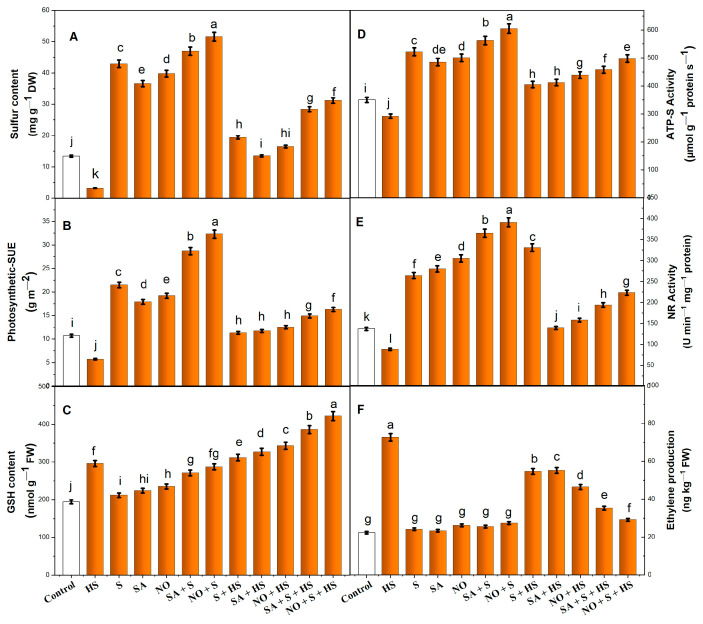
Sulfur content (**A**), photosynthetic sulfur use efficiency (p-SUE) (**B**), reduced glutathione (GSH) content (**C**), ATP-sulfurylase (ATP-S) activity (**D**), nitrate reductase (NR) activity (**E**) and ethylene production (**F**) in wheat (*Triticum aestivum* L.) cv. HD-3226 treated with 0.5 mM salicylic acid (SA) and/or 50.0 µM nitric oxide (NO)/2.0 mM SO_4_^−2^ (S) individually or in combination in presence or absence of heat stress (HS; 40 °C for 6 h daily for 15 days) at 30 days after germination. Data are presented as treatment mean (*n* = 4). Data followed by the same letter are not significantly different by the LSD test at *p* ≤ 0.05. DW, dry weight; FW, fresh weight.

HS decreased S content (−76.1%), p-SUE (−46.7%) and ATP-sulfurylase (ATP-S) activity (−16.5%) and increased GSH content (+52.5%) in comparison to the control (Figure 3A–D). Under non-stressed conditions, the application of SA, NO and S enhanced the S content, p-SUE, ATP-S activity and GSH content. However, the combined treatment of SA + S and NO + S exhibited the maximum increase. Under HS conditions, SA, NO and S caused a significant increase in the given attributes of the S assimilation and recovered the losses in S assimilation as a consequence of HS. Notably, the increases in S content, p-SUE, ATP-S activity and GSH content under the combined treatment of NO + S (132.8, 52.3, 42.2 and 117.5%) were more than SA + S (111.9, 39.5, 30.8 and 98.6%), suggesting the beneficial role of NO with S in HS tolerance (Figure 3A–D).

### 2.5. Application of NO, S and SA Maintains Nitrate Reductase (NR) Activity under HS

Heat treatment reduced NR activity by 35.7% compared to the control (Figure 3E). The individual supplementation of SA, NO or S to non-stressed plants enhanced NR activity, but it was lower than the value obtained in the combined treatment. SA + S and NO + S enhanced NR activity by 2.6-times and 2.8-times, respectively, compared to the control. Furthermore, the application of SA, NO or S to HS-exposed plants significantly reversed the inhibitory effects of HS on NR activity. However, combined SA + S and NO + S treatment recorded increases of 1.4- and 1.6-times, respectively, compared to the control (Figure 3E).

### 2.6. Impact of NO, S and SA on Ethylene Production under HS

The maximum evolution of ethylene was observed under HS, which showed an increase by 226.9% compared to the control plants (Figure 3F). Under the non-stressed condition, there was a marked reduction in ethylene production and there was no significant difference observed between the treatments and the control group when compared to the HS-exposed plants. Under HS, the individual treatments of SA and S were non-significant, whereas NO supply showed a reduction of 62.4%. Furthermore, the combined treatment of SA + S and NO + S witnessed a remarkable decrease in stress ethylene by 51.4 and 59.9%, respectively, compared to HS-exposed plants (Figure 3F).

### 2.7. Influence of SA Biosynthesis Inhibitor (AIP) and NO Biosynthesis Inhibitor (cPTIO) on Growth and Photosynthetic Parameters under HS

Under HS, leaf area and plant dry weight were reduced by 36.6 and 53.7%, respectively, as compared to the control. The combined application of NO and S, as well as SA and S, significantly alleviated the effect of HS and increased leaf area by 33.6 and 41.7% and plant dry weight by 37.5 and 32.5% in comparison to the control plants. Both cPTIO and AIP reduced the increase in growth characteristics observed with NO + S and SA + S during HS. Supplementation of cPTIO to the plants receiving NO + S under HS increased leaf area by 37.4% and plant dry weight by 31.2% in comparison to the control plants. Furthermore, compared to the control plants, AIP treatment to plants receiving SA + S under HS increased leaf area by 34.6% and plant dry weight by 25.0% (Table 3).

The treatment of HS decreased net photosynthesis, stomatal conductance, intercellular CO_2_ concentration, SPAD value and F_v_/F_m_ by 50.7, 30.6, 37.7, 37.8 and 29.2%, respectively, in comparison to the control (Table 3). In contrast, NO + S and SA + S increased net photosynthesis, stomatal conductance, intercellular CO_2_ concentration, SPAD value and F_v_/F_m_ by (40.1 and 34.0%), (27.0 and 23.6%), (45.2 and 39.6%), (27.3 and 22.6%) and (25.6 and 20.7%), respectively, under HS in comparison with the untreated control plants. cPTIO treatment to the plants receiving NO + S under HS showed an increase in the photosynthetic parameters compared to the control plants. Similarly, the photosynthetic parameters noted in SA + S + AIP under HS treatment were higher than control plants but lower than plants receiving SA + S and HS. When cPTIO was applied to plants receiving NO + S and HS, the photosynthetic parameters increased when compared to control plants but decreased when compared to plants receiving only NO + S and HS. AIP (SA biosynthesis inhibitor) and NO biosynthesis inhibitor (cPTIO) confirmed the influence of SA and NO on damage and defense markers under HS.

### 2.8. Influence of SA Biosynthesis Inhibitor (AIP) and NO Biosynthesis Inhibitor (cPTIO) on Oxidative Stress and Antioxidant Enzymes under HS

Treatment of HS led to a significant increases in H_2_O_2_ and TBARS content by (125.9%) and (220.7%), respectively, over the control (Table 4). The application of NO + S and SA + S mitigated the oxidative damage caused by HS by decreasing H_2_O_2_ and TBARS content by (31.8 and 22.9%) and (39.6 and 33.9%), respectively, as compared to the control. Application of cPTIO to plants receiving NO + S under HS showed a decrease in H_2_O_2_ and TBARS content by 20.7 and 30.1%, respectively, compared to the control plants. AIP along with the combined treatment of SA and S plus HS showed a decrease in H_2_O_2_ and TBARS content by only 15.5 and 26.4%, respectively, compared to the control plants.

Under HS, the treatment of NO + S and SA + S stimulated the increase in activity of antioxidant enzymes, namely, SOD by (196.3 and 172.8%), APX by (251.8 and 214.8%) and GR by (189.2 and 176.8%), respectively, relative to control plants (Table 4). When cPTIO was applied to NO plus S-treated plants under HS, the activity of these antioxidant enzymes was reduced compared to the NO plus S treatment under HS, but SOD activity increased by 156.2%, APX activity increased by 225.9% and GR activity increased by 168.5%, compared to control plants. A further reduction in the activity of the antioxidant enzymes occurred when AIP was applied to a combined NO and S treatment under HS, and the activity of SOD was increased by 132.6%, APX by 188.8% and GR by 152.0%, compared to control plants.

### 2.9. Influence of SA Biosynthesis Inhibitor (AIP) and NO Scavenger (cPTIO) on the Assimilation of S and N and the Evolution of Ethylene and Production of GSH under HS

Exposure to HS caused decreases in S content, p-SUE and ATP-S activity and an increase in GSH content by 42.8, 48.6, 26.9 and 6.69%, respectively, compared to the control (Table 5). Compared to the control, the application of NO + S and SA + S under HS increased S content by (63.2 and 57.1%), p-SUE by (36.6 and 25.6%), ATP-S activity by (142.3 and 130.7%), and GSH content by (50.3 and 47.1%), respectively. However, cPTIO (NO scavenger) application to HS-exposed *T. aestivum* supplied with NO + S decreased S content, p-SUE, ATP-S activity and GSH content when compared to HS-exposed *T. aestivum* receiving only NO + S. Furthermore, the addition of AIP to the plants supplemented with SA and S under HS showed a reduction in S content, p-SUE, ATP-S activity and GSH content compared to plants receiving only SA + S under HS.

Ethylene production was the highest in HS-exposed plants relative to control plants. When NO + S was applied to HS-exposed plants, it raised ethylene production by 166.6% when compared to control plants; however, it lowered ethylene production when compared to plants exposed to HS (Table 5). Under HS, SA with S lowered stress ethylene relative to heat-stressed plants but increased ethylene levels by 157.8% compared to control plants. Furthermore, cPTIO treatment of plants receiving NO and S under HS lowered ethylene evolution compared to plants receiving NO and S under HS; however, it increased ethylene levels compared to control plants. AIP treatment of plants that received SA and S under HS reduced ethylene evolution compared to plants that did not receive SA and S under HS but increased ethylene evolution compared to control plants.

### 2.10. Principal Component Analysis (PCA)

**Figure 4:**
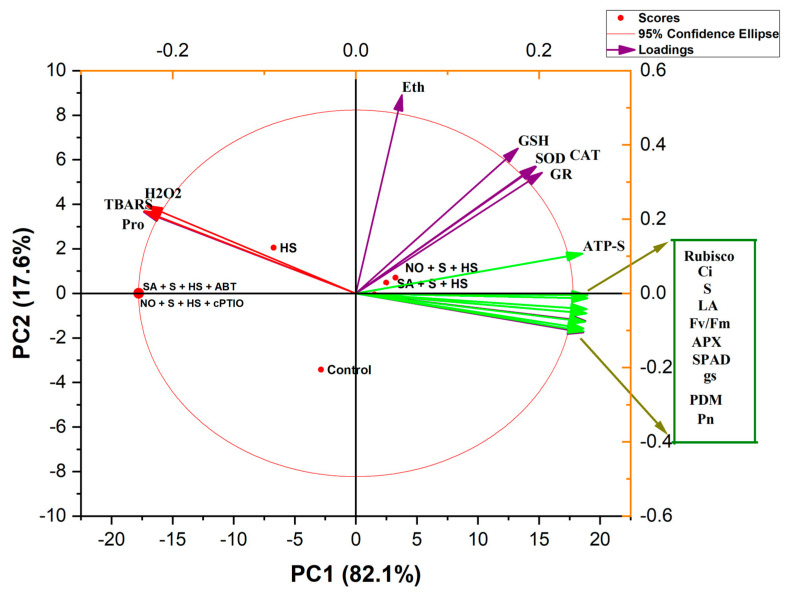
Principal component analysis (PCA) biplot for growth and physio-biochemical traits of *Triticum aestivum* plants. The treatments included control, heat stress (HS; 40 °C for 6 h daily for 15 days), NO + S + HS, SA + S + HS, NO + S + HS + cPTIO, SA + S + HS + ABD. The variables included ethylene (Eth), thiobarbituric acid reactive substances (TBARS), proline (Pro), hydrogen peroxide (H_2_O_2_), superoxide dismutase (SOD), glutathione reductase (GR), reduced glutathione (GSH), ATP-sulfurylase (ATP-S), Rubisco, leaf S (sulfur), net photosynthesis (Pn), stomatal conductance (gs), intercellular CO_2_ concentration (Ci), maximal PSII efficiency (F_v_/F_m_), plant dry mass (PDM) and leaf area (LA).

The scores of the PCA to evaluate the effects of NO and SA with S on *T. aestivum* under HS are presented in Figure 4. PC1 and PC2 accounted for 99.8% of the total variance in the dataset. Of them, PC1 contributed 82.1% and PC2 contributed 17.6% total variation. All the treatments were distributed successfully by the first two principal components (Figure 4). The HS treatment was distributed along with the oxidative stress biomarkers (H_2_O_2_, TBARS and proline content). The various observed parameters in the PCA biplot were divided into three clusters. Parameters such as H_2_O_2_, TBARS and proline content were close to the HS treatment. On the other hand, growth parameters (plant dry mass; PDM and leaf area; LA) and photosynthesis (net photosynthesis; Pn, stomatal conductance; gs, intercellular CO_2_ concentration; Ci, SPAD, maximum photochemical efficiency; F_v_/F_m_ and Rubisco activity) were close to the NO + S + HS and SA + S + HS treatments. Oxidative stress biomarkers and ethylene biosynthesis negatively correlated with plant growth and photosynthesis parameters. Enzymatic antioxidants (SOD, GR and GSH) and ethylene clustered between the oxidative stress parameters and plant growth and photosynthesis parameters suggesting their role in combating HS impacts. Therefore, the correlation biplot portrays a close association between NO + S and SA + S in the HS acclimation of *T. aestivum* plants (Figure 4).

## 3. Discussion

This study aimed to assess the major mechanisms underlying the role of exogenously-sourced NO (as sodium nitroprusside; SNP) and SA in the HS tolerance of *T. aestivum* in the presence of S supply. An effort was made hereunder to interpret and discuss the results obtained in all the experiments set out in the present study under three subheadings, namely, (i) screening of six *T. aestivum* cultivars for their p-SUE under HS; (ii) individual role of SA, NO and S in alleviating HS in *T. aestivum* cv. HD-3226 exhibiting the highest p-SUE; and (iii) confirmatory experimental results for ascertaining the involvement of SA and NO using SA biosynthesis inhibitor, AIP and NO scavenger, cPTIO, respectively, in mitigating the HS in *T. aestivum* cultivar HD-3226 exhibiting the highest p-SUE.

### 3.1. Screening of T. aestivum Cultivars for Their p-SUE under HS

Different cultivars of *T. aestivum* (HD-3059, HD-3090, HD-3226, HD-3237, HD-3271 and HI-1620) exhibited different responses towards p-SUE under HS. Notably, S is one of the key components in S-containing amino acids such as methionine (Met) and cysteine (Cys) and other S-containing compounds such as GSH [29]. Thus, the exhibition of the highest p-SUE in *T. aestivum* cultivar HD-3226 (among the *T. aestivum* cultivars considered herein) indicates its inherent capacity for activating the mentioned S compounds involving defense responses against elevated HS.

### 3.2. Individual and Combined Roles of SA, NO and S in Alleviating HS

In this study, HS had a deleterious effect on growth parameters (LA and PDM). However, the impact of HS on growth parameters were greatly alleviated by the individual application of NO or SA. The measured growth parameters were over-alleviated by treatment, with or without S. However, the combined application of SA or NO with S showed the maximum alleviation of HS and increased PDM and LA (Table 2). PDM and LA are two critical indicators of plant growth that are greatly influenced by adverse conditions. Additionally, the biomass and the content and yield of grain were also significantly affected during the HS in crop plants [5,30,31].

This study also considered gas exchange parameters along with chlorophyll fluorescence to understand the physiological insights into the impacts of HS as well as the effect of NO or SA with or without S on photosynthesis. In fact, photosynthesis is the only process that produces the sole basis of photosynthates and is highly prone to HS [32,33]. The structure of chloroplast is altered, and, eventually, the complexes comprising photosystems I-II (PS I and PS II), the electron quenchers, are inactivated under elevated HS and different light regimes [27,34]. The enhanced activity of chlorophyllase and chlorophyll-degrading peroxidase in response to HS was linked to decreased chlorophyll content [35]. The treatment of SNP can partially protect the photosynthesis rate and chlorophyll bleaching and maintain the photosynthetic content and thus the photosynthetic rate [36]. Moreover, the combined effect of NO or SA overcompensated the negative effect of HS. Findings obtained herein suggest that applying NO or SA with S under HS conditions favored S assimilation and the antioxidant system, which reduced the oxidative stress and in turn protected the chloroplast. In an earlier study, under salt stress, 0.1 mM SNP produced the most beneficial improvements, such as improving seed germination, the germination index, the vigor index, shoot height, taproot length, shoot biomass and root biomass [37].

This study showed that SNP with S improved chlorophyll content, gas exchange parameters and F_v_/F_m_ under HS. This was linked to the activation of the processes involved in the restoration of photosynthetic efficiency. Notably, SA is a ubiquitous phytohormone involved in the regulation of plant growth, photosynthesis and development in both normal and stressed conditions [17,18]. SA supply improved the decline in photosynthetic capacity, photosystem efficiency and, ultimately, the photosynthesis in *Arabidopsis thaliana* under high light conditions [38]. The decreases in net photosynthesis under HS alone may be related to a change in the balance of stomatal conductance and intercellular CO_2_ concentration [39]. As a result, when both the intercellular CO_2_ concentration and stomatal conductance decrease concurrently, the stomatal conductance limits the function of photosynthesis [40]. Photosynthesis, on the other hand, was found to increase *T. aestivum* supplied with NO or SA under no stress or HS. This could be due to the NO- or SA-mediated concurrent positive induction of stomatal conductance and intercellular CO_2_ concentration. The study of chlorophyll fluorescence may further illustrate the stress effects on plants, as well as the potential of plants to avert the consequences of adverse conditions [41]. The photosynthetic efficiency of the whole PSII and the maximal quantum yield of PSII are denoted by F_v_/F_m_ [42]. The results obtained in this study demonstrated that F_v_/F_m_ fell when plants were subjected to HS and was restored when plants were exposed to NO or SA with S in the presence of HS.

In the context of studies on the role of S and/or N, the photosynthetic performance of plants may be significantly reduced if they are deprived of either N or S [43,44]. In the current study, the application of NO or SA with S to HS-exposed *T. aestivum* enhanced NR activity and S uptake by elevating ATP-S activity to produce more S-containing compounds to be used in ROS metabolism for HS tolerance. ATP-S is the first rate-limiting enzyme in the S assimilation pathway and is also essential for Cys and GSH biosynthesis [45]. The results obtained herein support the role of ATP-S in maintaining the GSH pool essential for HS tolerance. The overexpression of ATP-S in Indian mustard exhibited a favorable effect on metal tolerance [46]. Moreover, the upregulation of the S assimilation pathway was reported to enhance plants’ sustenance under HS [19,47]. GSH levels in plant cells are maintained in a stable state under normal conditions; however, under stressed conditions, the equilibrium is disrupted, and the GSH pool is depleted to resist the stress. Interestingly, the supply of NO or SA with S to HS-exposed *T. aestivum* significantly enhanced GSH synthesis. This observation is in close conformity to the results obtained in HS-exposed *O*. *sativa* [5] and drought-exposed *B*. *napus* [48]. Notably, the involvement of S in HS tolerance is also possible via its influence on enhancing NR activity, N accumulation and Cys synthesis [49]. Furthermore, the production of ethylene, a gaseous signaling molecule, was also influenced by the application of SA and NO in the presence of S, which was found to be instrumental in the acclimation of *T. aestivum* to HS. In fact, the supplementation of SA or NO with S (via S-adenosyl methionine) interacted with the stress ethylene that was formed under HS, and optimized the ethylene level. In turn, stress ethylene and/or optimized ethylene levels regulated the antioxidant machinery, GSH synthesis, minimized oxidative stress and eventually protected *T. aestivum* against HS impacts. The mentioned conclusion is supported by the correlation biplot portraying a close association between NO + S, SA + S and NO + SA +S in the HS acclimation of *T. aestivum* (Figure 4). The role of ethylene in photosynthesis and growth under optimal conditions, and its involvement in stress acclimation via its interaction with other plant hormones are widely known [18,50,51,52].

### 3.3. Confirmatory Experimental Results for the Involvement of SA (Using AIP) and NO (Using cPTIO) in Mitigating HS

The current investigation also included the treatments of cPTIO and AIP, an NO scavenger and SA biosynthesis inhibitor, respectively, to ensure if the enhancements in growth, photosynthesis and antioxidant defense were attributable to NO and SA actions in the high p-SUE-exhibiting *T. aestivum* cv. HD-3226 under HS. The supply of AIP (SA biosynthesis inhibitor) or cPTIO (NO scavenger) to HS-exposed *T. aestivum* cv. HD-3226 brought increments in the oxidative markers (H_2_O_2_ and TBARS), significantly decreased the activity of SOD, GR and APX, and eventually decreased growth and photosynthesis. The responses of the aforementioned parameters with AIP or cPTIO treatment were on par with those obtained under HS alone and without SA, NO or S supply. Previously, significantly decreased CAT and POD activity was reported in AIP-supplied *Zea mays* under chilling stress [53]. In another study, the application of cPTIO confirmed the role of NO in HS tolerance [54].

## 4. Materials and Methods

### 4.1. Plant Culture and Treatments

Healthy seeds of wheat (*T. aestivum* L.) cultivars were procured from Indian Agricultural Research Institute, New Delhi, India. Before sowing, the seeds were surface sterilized with 0.01% HgCl_2_ followed by three times washing with deionized water. The sterilized seeds were soaked in distilled water for 12–24 h and incubated at 30 °C. These seeds were sown in earthen pots of 25 cm diameter filled with acid-washed sterilized sand as performed in previous experiments [18]. Thereafter, the pots were placed in the environmental growth chamber (Khera-Instruments, New Delhi, India) with the day/light regime of 16/8 h, photosynthetically active photon flux density of 200 μmol m^−2^ s^−1^ at plant level, the temperature of 25 °C in light and 18 °C in the dark with the relative humidity of 65 ± 5%. Initially, each pot contained ten seeds, which were sown. However, on the emergence of seedlings, thinning was conducted, and three seedlings were left in each pot. The plants were grown in sand culture, supplemented with Hoagland nutrients solution for the experiment. In the first experimentation, screening of *T. aestivum* cultivars, HD-3226, HD-3237, HD-3271, HD-3059, HD-3090 and HI-1620, was performed for their tolerance to HS (40 °C) based on photosynthesis, PDM and p-SUE. *T. aestivum* cultivars HD-3226 and HD-3090 exhibited high and low p-SUE, respectively. A set of plants were kept at 30 °C and taken as control plants, while another set of plants was treated with 40 °C (HS) for 6 h daily for 15 days and then allowed to recover at 30 °C and grown for the experimental period. The level of HS considered in this experiment was standardized previously [4].

The *T. aestivum* cultivar HD-3226 exhibiting the highest p-SUE was considered in the second experiment, which aimed to unveil the individual roles of sulfur (S; 2.0 mM SO_4_^−2^) and phytohormones (SA, 0.5 mM; and NO, 50.0 µM SNP) in alleviating HS (40 °C). The levels of S, SA and NO considered in this experiment were standardized previously [18,55]. S (2.0 mM SO_4_^•2^, 200 mL) was supplied as MgSO_4_, and Mg was uniformly maintained in all the treatments. Two hundred milliliters each of the two phytohormones, namely, SA, 0.5 mM and SNP, 50.0 µM, NO source were supplied on foliage along with the surfactant teepol (0.5%) on control and treatment plants.

The third experiment, which also considered HD-3226 (a high p-SUE exhibiting cultivar), was performed to confirm the involvement of two phytohormones, namely SA and NO, in mitigating HS impacts using SA biosynthesis inhibitor (i.e., 2-aminoindane-2-phosphonic acid; AIP, 0.5 mM) [53] and NO scavenger (2-4-carboxyphenyl-4,4,5,5-tetramethylimidazoline-1-oxyl-3-oxide; 100 µM cPTIO) [55,56]. *T. aestivum* cv. HD-3226 plants were supplied with and without S, SA and NO during HS in order to reveal the role of SA and NO in HS acclimation through the involvement of S.

In all the experiments, treatments were arranged in a random block design with four replicates (*n* = 4) for each treatment. Plants were sampled for the estimations at 30 days after germination (DAG).

### 4.2. Measurement of Growth Parameters, Photosynthetic Gas Exchange Parameters and Chlorophyll Content

*T. aestivum* plants were uprooted with proper care, cleaned properly to remove dirt particles and dried on blotting paper to determine dry weight. These plants were kept in an oven at 80 °C until they reached a constant weight and then weighed for their dry mass determination. The leaf area was measured using a leaf area meter (LA211 Systronics, New Delhi, India). Infrared gas analyzer (CID-340, Photosynthesis System, Bio Science, Camas, WA, USA) was used to determine photosynthetic gas exchange parameters (such as net photosynthetic rate, stomatal conductance and intercellular CO_2_ concentration) in fully expanded top young leaves. Photosynthetically active radiation (PAR) was 780 µmol m^−2^ s^−1^ while performing the experiment, and atmospheric CO_2_ concentrations were taken at 390 ± 5 µmol mol^−1^. Chlorophyll content was measured in the early morning using a SPAD chlorophyll meter (SPAD 502 DL PLUS, Spectrum Technologies, Plainfield, IL, USA) and expressed as SPAD values. The F_v_/F_m_ of the fully expanded second leaf from the top of the plant was determined using a chlorophyll fluorometer (Junior-PAM, Heinz Walz, GmbH, Effeltrich, Germany). The details are provided in Supplementary File S1.

### 4.3. Determination of H_2_O_2_ Content and Lipid Peroxidation

The method of Okuda et al. [57] was followed for the estimation of H_2_O_2_ content in fresh leaf tissues. The details of the method are given in Supplementary File S1. The concentration of TBARS was calculated adopting the method of Dhindsa et al. [58] in order to evaluate lipid peroxidation or membrane damage. The details are provided in Supplementary File S1.

### 4.4. Determination of Leaf S Content, Assays of Antioxidant Enzyme, NR and ATP-S Activity

The method of Chesnin and Yien [59] was followed to determine the leaf S content. The details are provided in Supplementary File S1.

Fresh leaf tissues (200 mg) were crushed in an ice-cold extraction buffer comprising potassium–phosphate buffer (100 mM, pH 7.0), 0.05% (*v*/*v*) Triton X-100 and 1% (*w*/*v*) polyvinylpyrrolidone (PVP). The homogenate was then centrifuged for 20 min at 4 °C at 15,000× *g*. The enzymatic activities were assayed in the clear supernatant obtained after centrifugation. APX extraction buffer with 2 mM ascorbate was used in the assays.

The inhibition of the photochemical reduction of nitro blue tetrazolium (NBT) was used to evaluate SOD activity in protein extracts, as described by [60,61]. The activity of APX was determined using the method of Nakano and Asada [62]. The GSH-dependent oxidation of nicotinamide adenine dinucleotide phosphate (NADPH) was measured at 340 nm to estimate GR activity, as described [63]. The details of the procedure are given in Supplementary File S1.

The leaf NR activity was assayed following the method of Kuo et al. [64] to prepare the enzyme extract. NR activity was measured by adopting the method of Nakagawa et al. [65] spectrophotometrically as the rate of nitrite production at 28 °C. The details of the procedure are given in Supplementary File S1.

ATP-S activity was assayed following the method of Lappartient and Touraine [66]. The details of the method are given in Supplementary File S1.

### 4.5. Determination of Photosynthetic SUE, GSH Content and Ethylene Production

Photosynthetic sulfur use efficiency was calculated by the ratio of net photosynthesis to S content per unit leaf area [5].

The method of Anderson [67] was followed to determine GSH content. The details of the method are given in Supplementary File S1.

Evolution of ethylene was analyzed following the procedure described by Fatma et al. [68]. The details of the method are given in Supplementary File S1.

### 4.6. Statistical Analysis

Data were analyzed statistically by using analysis of variance (ANOVA) by SPSS 17.0 for windows and presented as mean ± SE (*n* = 4), and the significance level at *p* < 0.05 was calculated using the least significant difference (LSD) test.

## 5. Conclusions

Among the six cultivars of *T. aestivum* screened for their heat tolerance, HD-3226 and HD-3090 showed the highest p-SUE and maximum heat tolerance, and the lowest p-SUE and minimum heat tolerance, respectively. Exogenous application of NO, SA and S modulated the stress-tolerance mechanisms when applied individually and also in combination with or without HS in the highest p-SUE exhibiting HD-3226. Additionally, NO and SA supply improved the photosynthesis machinery, but more prominently in the presence of S. Furthermore, SA and NO supplementation in the presence of S minimized HS-caused oxidative stress by maintaining a fine tuning among the antioxidant defense system components and ethylene production; this increased chlorophyll content, and eventually protected PS II activity under HS. The use of SA biosynthesis inhibitor (AIP) and NO scavenger (cPTIO) confirmed the involvement of SA and NO in HS tolerance in the presence of S.

## Figures and Tables

**Table 1:** Photosynthetic sulfur use efficiency (p-SUE), plant dry weight and net photosynthesis of wheat (*Triticum aestivum* L.) cultivars HD-3226, HD-3237, HD-3271, HD-3059, HD-3090 and HI-1620 grown in the presence or absence of heat stress (HS; 40 °C for 6 h daily for 15 days) at 30 days after germination.

Cultivar	Treatments	p-SUE (g m^−2^)	Plant Dry Weight (g plant^−1^)	Net Photosynthesis (μmol CO_2_ m^−2^ s^−1^)
HD-3226	Control	13.5 ± 0.6 ^a^	1.19 ± 0.05 ^a^	16.8 ± 0.8 ^a^
	HS	07.1 ± 0.3 ^d^	0.88 ± 0.04 ^g^	13.0 ± 0.6 ^d^
HD-3237	Control	12.9 ± 0.6 ^b^	1.13 ± 0.05 ^b^	14.5 ± 0.7 ^b^
	HS	06.4 ± 0.3 ^e^	0.82 ± 0.04 ^h^	10.8 ± 0.5 ^g^
HD-3271	Control	12.6 ± 0.6 ^bc^	1.11 ± 0.05 ^c^	13.6 ± 0.6 ^c^
	HS	06.0 ± 0.3 ^ef^	0.78 ± 0.03 ^hi^	09.9 ± 0.4 ^h^
HD-3059	Control	11.2 ± 0.5 ^cd^	1.05 ± 0.05 ^e^	11.7 ± 0.5 ^f^
	HS	05.1 ± 0.2 ^fg^	0.72 ± 0.03 ^ij^	08.2 ± 0.4 ^i^
HD-3090	Control	10.9 ± 0.5 ^cd^	1.01 ± 0.05 ^f^	11.1 ± 0.5 ^g^
	HS	04.8 ± 0.2 ^g^	0.68 ± 0.03 ^j^	07.1 ± 0.3 ^j^
HI-1620	Control	11.7 ± 0.5 ^c^	1.07 ± 0.05 ^d^	12.4 ± 0.6 ^e^
	HS	05.5 ± 0.2 ^f^	0.75 ± 0.03 ^i^	08.7 ± 0.4 ^i^

Data are presented as treatment mean (*n* = 4). Data followed by the same letter are not significantly different by the LSD test at *p* < 0.05.

**Table 2:** Chlorophyll content (SPAD value), net photosynthesis, stomatal conductance, intercellular CO_2_ concentration, leaf area, plant dry weight and maximum quantum efficiency of pigment system (PS) II of wheat (*Triticum aestivum* L.) cv. HD-3226 treated with 0.5 mM salicylic acid (SA) and/or 50.0 µM nitric oxide (NO)/2.0 mM SO_4_^−2^ (S) individually or in combination in the presence or absence of heat stress (HS; 40 °C for 6 h daily for 15 days) at 30 days after germination.

Treatments	Chlorophyll Content (SPAD Value)	Net Photosynthesis (µmol CO_2_ m^−2^ s^−1^)	Stomatal Conductance (mmol CO_2_ m^−2^ s^−1^)	Intercellular CO_2_ Concentration (µmol CO_2_ mol^−1^)	Leaf Area (cm^2^ Plant^−1^)	Plant Dry Weight (g Plant^−1^)	Maximum Quantum Efficiency of PS II
Control	33.7 ± 1.2 ^j^	10.7 ± 0.3 ^i^	403 ± 9.3 ^g^	237 ± 5.5 ^f^	24.7 ± 0.3 ^i^	0.87 ± 0.05 ^f^	0.80 ± 0.04 ^e^
HS	23.3 ± 0.9 ^k^	5.7 ± 0.2 ^j^	292 ± 7.2 ^h^	147 ± 3.7 ^h^	16.7 ± 0.2 ^j^	0.47± 0.03 ^g^	0.62 ± 0.05 ^f^
S	38.8 ± 1.2 ^g^	13.0 ± 0.5 ^f^	465 ± 11.2 ^de^	280 ± 6.2 ^de^	30.6 ± 0.3 ^f^	1.01 ± 0.06 ^e^	0.85 ± 0.03 ^cd^
SA	39.7 ± 1.2 ^f^	13.2 ± 0.6 ^ef^	470 ± 11.7 ^d^	289 ± 6.5 ^d^	32.3 ± 0.4 ^e^	1.03 ± 0.06 ^de^	0.88 ± 0.06 ^c^
NO	40.1 ± 1.3 ^e^	13.5 ± 0.6 ^e^	476 ± 12.2 ^cd^	315 ± 7.1 ^cd^	33.7 ± 0.4 ^de^	1.06 ± 0.07 ^d^	0.89 ± 0.30 ^c^
SA + S	41.9 ± 1.3 ^b^	14.7 ± 0.6 ^b^	487 ± 12.5 ^b^	330 ± 7.2 ^bc^	36.2 ± 0.4 ^b^	1.15 ± 0.13 ^b^	0.95 ± 0.05 ^b^
NO + S	42.7 ± 1.3 ^a^	15.3 ± 0.7 ^a^	493 ± 12.8 ^a^	355 ± 7.7 ^a^	38.1 ± 0.5 ^a^	1.18 ± 0.13 ^a^	0.97 ± 0.09 ^a^
S + HS	35.2 ± 1.2 ^i^	11.4 ± 0.4 ^g^	425 ± 9.7 ^f^	208 ± 6.3 ^g^	26.3 ± 0.2 ^h^	0.95 ± 0.06 ^e^	0.82 ± 0.07 ^d^
SA + HS	35.5 ± 1.2 ^i^	11.6 ± 0.4 ^g^	432 ± 10.2 ^ef^	225 ± 5.3 ^fg^	26.8 ± 0.2 ^h^	0.97 ± 0.06 ^e^	0.82 ± 0.05 ^d^
NO + HS	36.2 ± 1.2 ^h^	12.8 ± 0.4 ^h^	443 ± 11.3 ^e^	260 ± 5.7 ^e^	27.5 ± 0.2 ^g^	1.03 ± 0.06 ^de^	0.85 ± 0.02 ^cd^
SA + S + HS	40.7 ± 1.2 ^d^	13.9 ± 0.5 ^d^	480 ± 12.1 ^c^	281 ± 5.8 ^c^	34.3 ± 0.4 ^d^	1.10 ± 0.11 ^de^	0.92 ± 0.03 ^bc^
NO+ S + HS	41.2 ± 1.3 ^c^	14.3 ± 0.6 ^c^	483 ± 12.2 ^bc^	312 ± 6.8 ^b^	35.7 ± 0.4 ^c^	1.12 ± 0.11 ^c^	0.94 ± 0.07 ^b^

Data are presented as treatment mean (*n* = 4). Data followed by the same letter are not significantly different by the LSD test at *p* ≤ 0.05.

**Table 3:** Chlorophyll content (SPAD value), net photosynthesis, stomatal conductance, intercellular CO_2_ concentration, leaf area, plant dry weight and maximum quantum efficiency of pigment system (PS) II in wheat (*Triticum aestivum* L.) cv. HD-3226 treated with 0.5 mM salicylic acid (SA) or 50.0 µM nitric oxide (NO) together with 2.0 mM SO_4_^−2^ and/or 0.5 mM AIP and/or 100 µM cPTIO in presence or absence of heat stress (HS; 40 °C for 6 h daily for 15 days) at 30 days after germination.

Treatments	Chlorophyll Content (SPAD value)	Net Photosynthesis (µmol CO_2_ m^−2^ s^−1^)	Stomatal Conductance (mmol CO_2_ m^−2^ s^−1^)	Intercellular CO_2_ Concentration (µmol CO_2_ mol^−1^)	Leaf Area (cm^2^ Plant^−1^)	Plant Dry Weight (g Plant^−1^)	Maximum Quantum Efficiency of PS II
Control	30.4 ± 1.0 ^d^	13.2 ± 0.6 ^d^	385 ± 19.2 ^e^	212 ± 10.6 ^d^	27.3 ± 1.3 ^e^	0.80 ± 0.04 ^d^	0.82 ± 0.04 ^e^
HS	18.9 ± 0.9 ^e^	6.5 ± 0.3 ^e^	267 ± 13.3 ^f^	132 ± 06.6 ^e^	17.3 ± 0.8 ^f^	0.37 ± 0.01 ^e^	0.58 ± 0.02 ^f^
SA + S + HS	37.3 ± 1.8 ^b^	17.7 ± 0.8 ^b^	476 ± 23.8 ^b^	296 ± 14.8 ^b^	36.5 ± 1.8 ^c^	1.06 ± 0.05 ^b^	0.99 ± 0.05 ^c^
NO + S + HS	38.7 ± 1.9 ^a^	18.5 ± 0.9 ^a^	489 ± 24.4 ^a^	308 ± 15.4 ^a^	38.7 ± 1.9 ^a^	1.10 ± 0.05 ^a^	1.03 ± 0.05 ^a^
SA + S + HS + AIP	35.7 ± 1.7 ^c^	16.3 ± 0.8 ^cd^	458 ± 22.9 ^d^	284 ± 14.2 ^c^	34.6 ± 1.7 ^d^	1.00 ± 0.05 ^c^	0.96 ± 0.05 ^d^
NO + S + HS + cPTIO	37.5 ± 1.8 ^b^	16.8 ± 0.8 ^c^	472 ± 23.6 ^c^	291 ± 14.5 ^bc^	37.4 ± 1.8 ^b^	1.05 ± 0.05 ^b^	1.01 ± 0.05 ^b^

Data are presented as treatment mean (*n* = 4). Data followed by the same letter are not significantly different by the LSD test at *p* < 0.05. AIP, 2-aminoindane-2-phosphonic acid; cPTIO, 2-4-carboxyphenyl-4,4,5,5-tetramethylimidazoline-1-oxyl-3-oxide.

**Table 4:** Content of H_2_O_2_ and TBARS (thiobarbituric acid reactive substance), and the activity of SOD (superoxide dismutase), APX (ascorbate peroxidase) and GR (glutathione reductase) of wheat (*Triticum aestivum* L.) cv. HD-3226 treated with 0.5 mM salicylic acid (SA) or 50.0 µM nitric oxide (NO) together with 2.0 mM SO_4_^−2^ and/or 0.5 mM AIP and/or 100 µM cPTIO in presence or absence of heat stress (HS; 40 °C for 6 h daily for 15 days) at 30 days after germination.

Treatments	H_2_O_2_ Content	TBARS Content	SOD Activity	APX Activity	GR Activity
	(nmol g^−1^ FW)		(U min^−1^ mg^−1^ Protein)		
Control	13.5 ± 0.6 ^b^	5.3 ± 0.2 ^b^	7.22 ± 0.3 ^f^	2.7 ± 0.1 ^f^	2.42 ± 0.1 ^e^
HS	30.5 ± 1.5 ^a^	17.1 ± 0.8 ^a^	12.90 ± 0.6 ^e^	5.3 ± 0.2 ^e^	4.22 ± 0.2 ^d^
SA + S + HS	10.4 ± 0.5 ^e^	3.5 ± 0.1 ^d^	19.70 ± 0.9 ^b^	8.5 ± 0.4 ^c^	6.70 ± 0.3 ^b^
NO + S + HS	9.2 ± 0.4 ^f^	3.2 ± 0.1 ^de^	21.40 ± 1.0 ^a^	9.5 ± 0.4 ^a^	7.00 ± 0.3 ^a^
SA + S + HS + AIP	11.4 ± 0.5 ^c^	3.9 ± 0.1 ^c^	16.80 ± 0.8 ^d^	7.8 ± 0.3 ^d^	6.10 ± 0.3 ^c^
NO + S + HS + cPTIO	10.7 ± 0.5 ^d^	3.7 ± 0.1 ^cd^	18.50 ± 0.9 ^c^	8.8 ± 0.4 ^b^	6.50 ± 0.3 ^bc^

Data are presented as treatment mean (*n* = 4). Data followed by the same letter are not significantly different by the LSD test at *p* < 0.05. AIP, 2-aminoindane-2-phosphonic acid; cPTIO, 2-4-carboxyphenyl-4,4,5,5-tetramethylimidazoline-1-oxyl-3-oxide.

**Table 5:** Activity of ATP-sulfurylase (ATP-S), sulfur content, photosynthetic sulfur use efficiency (p-SUE), reduced glutathione (GSH) content and ethylene production of wheat (*Triticum aestivum* L.) cv. HD-3226 treated with 0.5 mM salicylic acid (SA) or 50.0 µM nitric oxide (NO) together with 2.0 mM SO_4_^−2^ and/or 0.5 mM AIP and/or 100 µM cPTIO in presence or absence of heat stress (HS) at 30 days after germination.

Treatments	ATP-S Activity (µmol g^−1^ protein s^−1^)	Sulfur Content (mg g^−1^ DW)	p-SUE	GSH Content (nmol g^−1^ FW)	Ethylene Production (ng kg^−1^ FW s^−1^)
Control	2.6 ± 0.3 ^d^	4.9 ± 0.2 ^d^	10.9 ± 0.5 ^e^	284 ± 14.2 ^f^	24.7 ± 1.2 ^e^
HS	1.9 ± 0.1 ^e^	2.8 ± 0.1 ^e^	05.6 ± 0.2 ^f^	303 ± 15.1 ^e^	70.8 ± 3.5 ^a^
SA + S + HS	6.0 ± 0.3 ^b^	7.7 ± 0.3 ^bc^	13.7 ± 0.6 ^a^	418 ± 20.9 ^b^	63.7 ± 3.1 ^cd^
NO + S + HS	6.3 ± 0.3 ^a^	8.0 ± 0.4 ^a^	14.9 ± 0.7 ^a^	427 ± 21.3 ^a^	65.8 ± 3.2 ^b^
SA + S + HS + AIP	5.7 ± 0.2 ^c^	7.3 ± 0.3 ^c^	12.6 ± 0.6 ^d^	371 ± 18.5 ^d^	60.3 ± 3.0 ^d^
NO + S + HS + cPTIO	6.1 ± 0.3 ^bc^	7.8 ± 0.3 ^c^	13.4 ± 0.6 ^c^	387 ± 19.3 ^c^	63.9 ± 3.1 ^c^

Data are presented as treatment mean (*n* = 4). Data followed by same letter are not significantly different by LSD test at *p* < 0.05. AIP, 2-aminoindane-2-phosphonic acid; cPTIO, 2-4-carboxyphenyl-4,4,5,5-tetramethylimidazoline-1-oxyl-3-oxide; DW, dry weight; FW, fresh weight.

## Data Availability

The data presented in this study are available in the graphs provided in the manuscript.

## Supplementary Materials

The following are available online at https://www.mdpi.com/article/10.3390/plants11223131/s1, Material and Methods details.
