# Supplementary material for: Nitric Oxide and Salicylic Acid Regulate Glutathione and Ethylene Production to Enhance Heat Stress Acclimation in Wheat Involving Sulfur Assimilation
Source: Plants (Basel). 2022 Nov 16;11(22):3131. doi: 10.3390/plants11223131 (PMC9697704; doi:10.3390/plants11223131)
Supplement: Supplementary file 1 [file plants-11-03131-s001.zip › Supplementary File.pdf]

Nitric Oxide and Salicylic Acid Regulate Sulfur-Assimilation to Enhance Heat Stress-Acclimation in Wheat

Faisal Rasheed[1], Iqbal R. Mir[1], Zebus Sehar[1], Mehar Fatma[1], Harsha Gautam[1], Sheen Khan[1], Naser A. Anjum[1], Asim Masood[1], Adriano Sofo[2,*] and Nafees A. Khan[1,*]

[1]Plant Physiology and Biochemistry Laboratory, Department of Botany, Aligarh Muslim University, Aligarh-202002, India
[2]Department of European and Mediterranean Cultures: Architecture Environment, Cultural Heritage, University of Basilicata, 75100 Matera, Italy
[*]Correspondence: adriano.sofo@unibas.it (A.S.); naf9.amu@gmail.com (N.A.K.)

Details of Methods

PSII activity

Plants were kept in dark conditions for 30 minutes to get minimal fluorescence (F_o) and maximum fluorescence (F_m). The F_o was measured using weak measuring pulses (125µmol m^{-2} s $^{-1}$), and the F_m was obtained using a saturating pulse (720 µmol m^{-2} s $^{-1}$). The difference between F_o and F_m was used to calculate variable fluorescence (F_v). The ratio of variable fluorescence to peak fluorescence was used to indicate PS II's quantum yield efficiency.

Determination of H_2O_2 content and lipid peroxidation

Leaf tissues (500 mg) were homogenized in ice-cold 200 mM $HClO_4$ for determination of H_2O_2 content. The homogenate was centrifuged at 1200 × *g* for 10 minutes followed by the neutralization of $HClO_4$ of the supernatant with 4 M KOH. Further centrifugation was done at 500 × *g* for 3 minutes to remove the insoluble $KClO_4$. The reaction mixture contained 1 ml of the eluate, 400 µl of 12.5 mM 3-(dimethylamino) benzoic acid in 0.375 M phosphate buffer (pH 6.5), 80µl of 3-methyl-2-benzothiazoline hydrazone, and 20µl of peroxidase in a final volume of 1.5 ml (0.25 Unit). At 25 °C, peroxidase was added to initiate the reaction, and the absorbance was recorded at 590 nm.

Leaf tissues (500 mg) were powdered in 0.25 % 2-thiobarbituric acid in 10% trichloroacetic acid for the estimation of TBARS content, then heated for 30 minutes at 95 °C and cooled fast on an ice bath. After that, the samples were centrifuged for 10 minutes at 10,000 x *g*. A 1 ml sample of the supernatant was treated with 4 ml of 20% trichloroacetic acid containing 5% thiobarbituric acid. At 532 nm, the colour intensity was measured.

Estimation of leaf S content

Digestive tube (75 ml) was filled with oven-dried leaf powder (100 mg). To the digestion tube, 7.5 mg of selenium dioxide was added as a catalyst, and the acid combination (4 ml) was made using concentrated HNO_3 and 60 % $HClO_4$ in an 85:1 (v/v) ratio. The combination was digested until it yielded a colourless solution, and then double distilled water was used to increase the volume to 75 ml. The content of the tube was filtered to the potential interference of silica. For turbidity development, a 5 ml aliquot was transferred from the digestive tube to a 25 ml-volumetric flask. Turbidity development was started by adding 2.5 ml gum acacia (0.25%) solution and 1.0 g $BaCl_2$ (sieved through 40–60 mm filter). To this, 25 ml DDW was added, and the flask was shaken well to completely dissolve $BaCl_2$. The turbidity was allowed to grow for 2 minutes. Within 10 minutes of turbidity growth, the values were recorded at 415 nm. After each set of determinations, a blank was run at the same time, and calculations were made appropriately.

SOD activity

The enzyme-containing extract was combined with 5.0 ml of reaction mixture comprising 5 mM HEPES (pH 7.6), 0.1 mM EDTA, 50 mM Na_2CO_3 (pH 10.0), 13 mM methionine, 0.025 % (v/v) Triton X-100, 63 μmol NBT, and 1.3 μmol riboflavin. The reactants were then exposed to strong light (360 μmol m^{-2} s^{-1}) for 15 minutes while a control was left unilluminated for background absorbance adjustment. A unit of SOD is defined as the quantity of enzyme required to inhibit NBT decrease by 50% (as measured by the absorbance at 560 nm).

APX activity

Phosphate buffer (50 mM, pH 7.0), 0.1 mM EDTA, 0.5 mM ascorbate, 0.1 mM H_2O_2, and enzyme extract were added to a 1.0 mL assay mixture, which was measured at 290 nm for 1 minute with a spectrophotometer. As soon as the reaction began, there was a drop in absorbance (i.e., upon the addition of H_2O_2). When calculating APX activity, a 2.8 mM^{-1} cm^{-1} extinction coefficient was employed. At 25 °C, one unit of APX is the quantity required to degrade one μmol of substrate per minute.

GR activity

Phosphate buffer (25 mM, pH 7.8), 0.5 mM GSSG, 0.2 mM NADPH, and the enzyme extract were included in the reaction mixture (3.0 ml). When GSSG was added, the reaction began, and a declining trend in absorbance was immediately seen. When measuring GR activity, a 6.2 mM^{-1} cm^{-1} extinction coefficient was utilised. At 25 °C, one unit of enzyme is defined, as the quantity required to breakdown one μmol of NADPH per minute.

NR activity

Leaf nitrate reductase activity was measured in fresh leaves by preparing the enzyme extract. Leaf tissues were frozen in liquid N2 and ground with the help of mortar and pestle and then stored at −80 °C. The powder was thawed for 10 min at 4 °C and homogenised in 250 mM Tris HCl buffer (pH 8.5), containing 10 mM cysteine, 1.0 mM EDTA, 20 mM FAD, 1.0 mM DTT, and 10% (v/ v) glycerol. It was followed by centrifugation at 10,000× g for 30 min at 4 °C. Nitrate reductase activity was measured by spectrophotometrically as the rate of nitrite production at 28 °C (1984). The assay mixture contained KNO3 (10 mM), HEPES (0.065 M pH 7.0), NADH (0.5 mM) in phosphate buffer (0.04 mM, pH 7.2) and the enzyme extract in a final volume of 1.5 ml. The reaction was started by the addition of NADH. After 15 min of incubation, the reaction was terminated by the addition of 1.0 ml of 1.0 N HCl solution containing 1% sulphanilamide followed by the addition of 1.0 ml of 0.02% aqueous N-1-napthylethylene-di-aminedihydrochloride. The absorbance was recorded at 540 nm after 10 m

ATP-S activity

The homogenizing buffer comprised of Na_2EDTA (10 mM), Tris-HCl (20 mM; pH 8.0), dithiothreitol (DTT; 2 mM), and polyvinyl pyrrolidone (PVP; 0.01 g ml^{-1}) with a tissue to buffer ratio of 1:4 (w/v). The homogenate was centrifuged for 10 minutes at 4 °C at 20,000 x *g*. The resulting supernatant was used in an ATP-S test in vitro. The production of pyrophosphate in the presence of molybdate was used to measure ATP-S activity. To start the reaction, 0.1 ml of the extract was added to 0.5 ml of the reaction mixture, which included Na_2MoO_4 (5 mM), Na_2ATP (2 mM), $MgCl_2$ (7 mM), and sulphate-free inorganic pyrophosphate (0.032-unit ml^{-1}) in Tris-HCl buffer (80 mM; pH 8.0). Another aliquot of the same extract was treated with the same reaction mixture but without the Na_2MoO_4. Samples were incubated at 37 °C for 15 minutes. A spectrophotometer set to 660 nm was used to measure the amount of phosphate produced in the samples. A standard curve built from known PO_4^{3-} concentrations was used to determine ATP-S activity.

GSH content

Under cold conditions, fresh leaf tissues (500 mg) were homogenised in 2.0 ml of 5% sulphosalicylic acid. The homogenate was centrifuged for 10 minutes at 10000 × *g*. Phosphate buffer (0.6 ml; 100 mM, pH 7.0) and 40 µl of 5-dithiobis-2-nitrobenzoic acid (DTNB) were added to 0.5 ml of supernatant. The absorbance was measured at 412 nm after 2 minutes.

Ethylene production

A chopped leaf material of 0.5 g was placed in damp paper-lined 30 ml size of tubes. These tubes were air tight with secure rubber covers to avoid any leakage of ethylene from the tissue, and they were exposed to sunlight for 2 h under the same environmental conditions as used for normal plant growth. About 1-ml gas sample were withdrawn from the tubes using a hypodermic needle and analysed on a gas chromatograph (Nucon 5700, Nucon New Delhi, India). Gas chromatograph was equipped with a 1.8-m Porapack™ N (80–100 mesh) column, a flame ionisation detector for gas chromatography, and a data station. The mobile phase was taken as nitrogen. The flow rate of nitrogen, hydrogen, and oxygen were kept as 30, 30, and 300 ml min^{-1}, respectively. The constant temperature of the detector was placed at 150 °C. Ethylene was estimated by its retention time and quantified by comparing it to peaks from ethylene standards concentration.
